# Mind your tag in single-molecule measurements

**DOI:** 10.1016/j.crmeth.2023.100623

**Published:** 2023-10-25

**Authors:** Raquel Merino Urteaga, Taekjip Ha

**Affiliations:** 1 Program in Cellular and Molecular Medicine, Boston Children’s Hospital, Boston, MA, USA; 2 Department of Pediatrics, Harvard Medical School, Boston, MA, USA; 3 Department of Biology, Johns Hopkins University, Baltimore, MD, USA; 4 Howard Hughes Medical Institute, Boston, MA, USA

## Abstract

In this issue of *Cell Reports Methods*, Molina and colleagues use *in vitro* single-molecule DNA flow-stretching to demonstrate the severe effects of appending a short lysine-cysteine-lysine (KCK) tag on the *Bacillus subtilis* ParB protein. This assay could be further utilized to evaluate the impact of other tags on DNA-binding proteins.

## Main text

**Figure 1:**
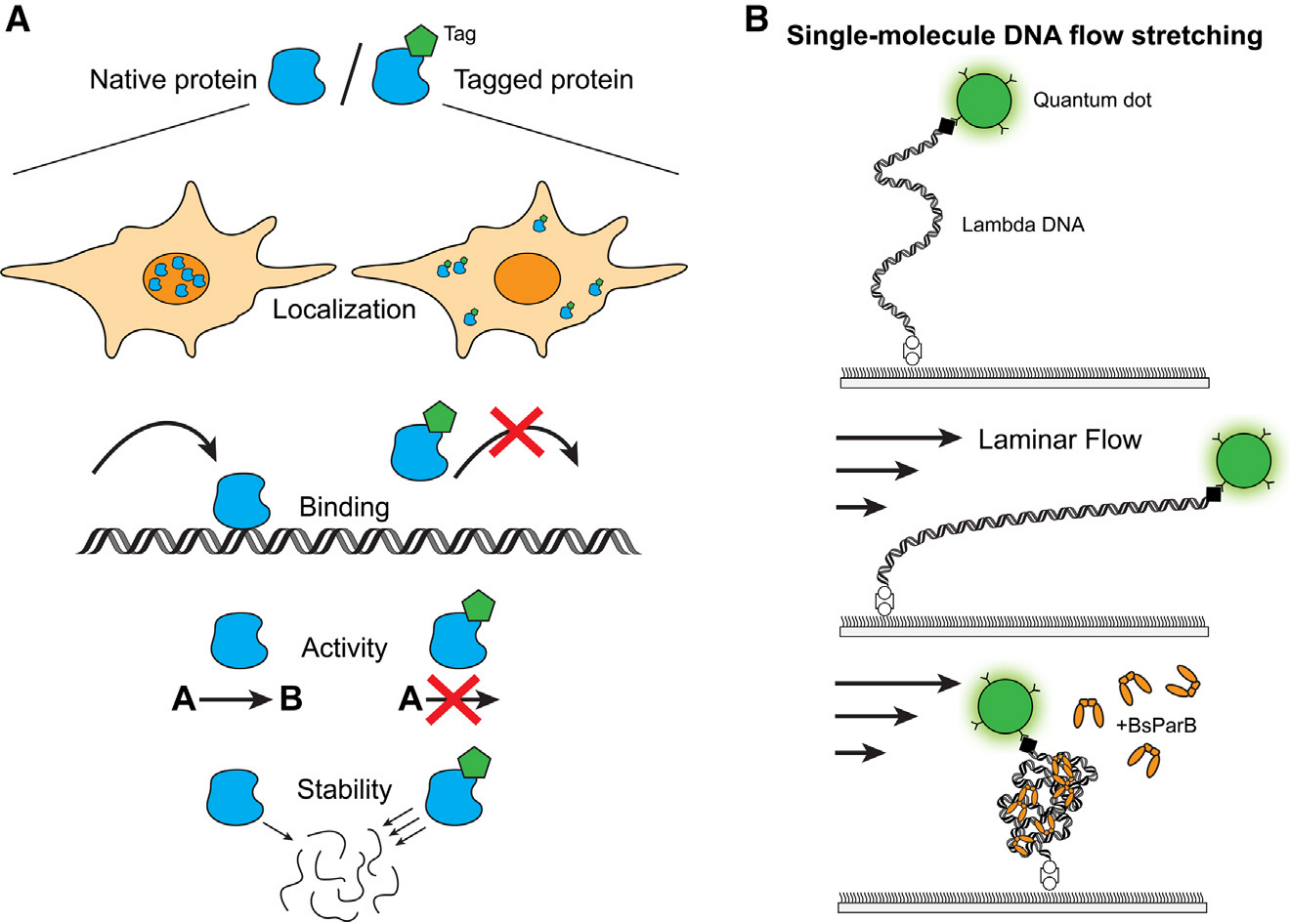
Possible effects of a tag on target proteins and schematic of single-molecule DNA flow stretching (A) Tags can structurally alter protein functions, causing mislocalization, disruption of protein binding and activity, as well as decreased protein stability and degradation of misfolded intermediates. (B) *In vitro* single-molecule characterization of DNA compaction by ParB. Quantum dot end labeled lambda DNA is tethered to microfluidic flow channel. DNA is stretched upon application of hydrodynamic flow. Incorporation of ParB compacts the stretched DNA, likely by bridging distant segments of DNA through ParB dimers and oligomers.

Protein tagging can dramatically alter the integrity of a labeled biomolecule, compromising its native properties. A tag can potentially introduce undesired perturbations that include changes on the expression level, stability, activity, and localization (Figure 1A). Previous studies have pointed out the adverse influence of some tags on protein activity. For instance, Huang et al. shed light on the multiple potential problems in the use of mCherry to study lysosome proteins.^1^ Another study addressed how epitope tags linked to different proteins can significantly alter their expression level in *Saccharomyces cerevisiae*.^2^ Given that protein tagging is extensively used in a wide range of biological tools, from affinity purification to fluorescence microscopy, a careful functional study of the labeled protein is crucial in order to select a suitable tag and minimize any unwanted alteration on the protein traits.

Single-molecule techniques have allowed us to characterize the dynamics of individual biomolecules over time and enabled researchers to register distinct subpopulations. The visualization of these biomolecules can be achieved through fluorescent tagging where probes are specifically linked to a target biomolecule and their spectroscopic behavior gives insights into complex biophysical properties. A range of single-molecule approaches have been developed to define protein and nucleic acids kinetics,^3^^,^^4^ conformation,^5^^,^^6^ stoichiometry,^7^ and function.^6^^,^^8^ However, each application requires a suitable label that must display good photophysical characteristics and minimal modification to the biomolecule’s properties. Therefore, the design of sensitive methods is required to determine whether a tag causes any adverse effect. In this issue of *Cell Reports Methods*, Molina et al.^9^ utilize single-molecule DNA flow-stretching to assess the impact of a lysine-cysteine-lysine (KCK) tag, commonly used for fluorescent labeling via the thiol moiety in the cysteine, on the DNA-binding protein ParB. The positive charges surrounding the cysteine are considered to be beneficial in terms of labeling efficiency.

The ParAB-parS partition system controls the segregation of newly replicated chromosomes and plasmids in bacterial cells. It is composed of an ATPase motor protein, ParA, responsible for the movement of chromosomes to the distal pole of the cell; a DNA-binding protein ParB; and its target sequence *parS* located in the vicinity of the replication origin. ParB nucleates around the *parS* sequence and spreads on to adjacent DNA, more than 10,000 base pairs, ultimately forming big nucleoprotein complexes. These interactions result in dramatic compaction of the DNA via bridging and looping.^10^ Aiming to unravel the mechanism involved in ParB DNA condensation, previous studies have used a KCK-tag on the ParB protein to chemically conjugate a Cy3 fluorescent dye.^11^ Molina et al.^9^ now demonstrate that the insertion of a KCK-tag on ParB alters its properties *in vitro*.

The methodology used by Molina et al.^9^ centers on stretching DNA with a stretching force generated by the hydrodynamic drag of a buffer flow. The authors use this assay to measure the rate of lambda DNA condensation induced by the *B. subtilis* ParB (ParB) (Figure 1B). In the absence of added nucleotide, both unlabeled wild-type (WT) ParB and KCK-ParB, ParB tagged with KCK at its N terminus, displayed rapid DNA compaction with comparable rates, as measured by the movement of a quantum dot attached to the DNA end. The presence of 1 mM CTP or 1 mM CTPγS, a non-hydrolyzable CTP analog, markedly reduced DNA compaction rate of untagged ParB by 39-fold and 149-fold, respectively. However, the nucleotides induced only a 2- to 3-fold decrease in DNA compaction rate when KCK-ParB was used. Hence, appending a KCK-tag to ParB appears to render the protein much less responsive to CTP.

The authors further evaluated the activity of KCK-tag ParB on lambda DNA containing one *parS* site located in the middle (1-parS DNA). As with lambda DNA without *parS*, CTP and CTPγS diminished the rate of DNA compaction induced by ParB, and the KCK-tag mitigated the decrease in 1-parS DNA compaction caused by CTP. Previous studies have investigated the role of CTP in the interaction between *parS* DNA and ParB proteins.^12^ Utilizing optical and magnetic tweezers, the authors had visualized the specific binding of ParB to *parS* sites and showed that CTP can significantly enhance DNA compaction. The findings are seemingly conflicting with the results obtained by Molina et al.^9^ here; however, the discrepancies could be explained by the experimental setting used in each investigation. First, the different approaches used by each group render distinct conditions that can influence DNA loop formation. The DNA flow-stretching assay in Molina et al.^9^ generates lower tension on the free ends of the DNA, while optical trap experiments in the previous study subjects DNA to a uniform tension along the DNA. Second, the DNA substrate here contains one *parS* sequence inserted on the lambda DNA, while de Balaguer et al.^12^ utilize DNA with multiple *parS* sites (39 and 13 repeats). The presence of many *parS* sequences on the latter could be emphasizing the effects of ParB binding to specific sequences, which is known to be influenced by CTP. It is possible that CTP makes ParB binding to DNA more sequence specific, and this communication between CTP binding and sequence-specific DNA binding is altered by the KCK-tag.

Molina et al.^9^ also examined the effect of the KCK-tag on the R80A ParB mutant, which is incapable of compacting DNA.^11^ Regardless of the presence of CTP and whether it is with either lambda or 1-parS DNA, the insertion of a KCK-tag on ParB(R80A) greatly increased compaction rates. How does the KCK-tag increase DNA compaction by this mutant? To directly address this question, they performed DNA flow-stretching using KCK-tagged ParB(R80A) nonspecifically labeled with Cy3 fluorophores. Looking at the integrated Cy3 fluorescence intensity over time, Molina et al.^9^ could attribute improved DNA compaction by the tagged protein to its greater loading onto the DNA. Therefore, the KCK-tag appears to nullify the DNA binding defect induced by the mutation.

The authors hypothesized that the increased nonspecific DNA binding of KCK-tagged proteins and the resulting increase in compaction activities are due to the positive charges on the two lysine residues, interacting electrostatically with negatively charged DNA. Indeed, they confirmed that a negatively charged glutamic acid-cysteine-glutamic acid (ECE) tag inserted to the N terminus of ParB has the opposite effect, slowing down DNA compaction relative to the untagged protein. Another question was whether the artifact caused by the KCK-tag would disappear when the tag is fluorescently labeled. This was not the case according to their experiments using KCK-ParB labeled with Alexa 647 at the cysteine residue in the tag.

Based on the *in vitro* alteration brought on by the KCK-tag, the authors questioned whether this label affects ParB localization and spreading *in vivo*. Using fluorescence microscopy on GFP fusions to KCK-tagged ParB(WT) and ParB(R80A), they demonstrated that the cellular localization of the KCK-tagged variants is similar to their respective untagged counterparts. To test the possibility that the GFP tag may negate the effect of the KCK-tag in the live cell imaging studies, they performed chromatin immunoprecipitation assays using anti-ParB antibodies and found that KCK-ParB (without GFP) spreads to the surroundings of a *parS* site, similar to untagged ParB. Additionally, both ParB(R80A) and KCK-ParB(R80A) were restricted to the *parS* site without spreading outward. Therefore, KCK tagging does not perturb ParB interactions with DNA when tested *in vivo*.

An important lesson we can derive from this study is that showing that protein tagging does not alter *in vivo* activities does not necessarily guarantee minimal functional perturbations under other conditions such as *in vitro* and biochemical reconstitution. In cells, there may be factors that mitigate the negative effects of tags. Therefore, this study highlights the importance of performing a comparative analysis of tagged and untagged proteins under each assay condition used.
